# Joint EANM/EANO/RANO/SNMMI practice guideline/procedure standards for diagnostics and therapy (theranostics) of meningiomas using radiolabeled somatostatin receptor ligands: version 1.0

**DOI:** 10.1007/s00259-024-06783-x

**Published:** 2024-06-20

**Authors:** Nathalie L. Albert, Matthias Preusser, Tatjana Traub-Weidinger, Nelleke Tolboom, Ian Law, Joshua D. Palmer, Eric Guedj, Julia Furtner, Francesco Fraioli, Raymond Y. Huang, Derek R. Johnson, Christophe M. Deroose, Ken Herrmann, Michael Vogelbaum, Susan Chang, Joerg-Christian Tonn, Michael Weller, Patrick Y. Wen, Martin J. van den Bent, Antoine Verger, Jana Ivanidze, Norbert Galldiks

**Affiliations:** 1 Department of Nuclear Medicine, LMU Hospital, LMU Munich, Marchioninistr. 15, 81377 Munich, Germany; 2 Department of Neurological Surgery, University of California at San Francisco, San Francisco, CA USA; 3 Nuclear Medicine and Molecular Imaging, Department of Imaging and Pathology, University Hospitals Leuven, KU Leuven, Leuven, Belgium; 4 Institute of Nuclear Medicine, University College London (UCL), London, UK; 5 Research Center for Medical Image Analysis and Artificial Intelligence (MIAAI), Faculty of Medicine and Dentistry, Danube Private University, 3500 Krems, Austria; 6 Institut Fresnel, Nuclear Medicine Department, APHM, CNRS, Timone Hospital, CERIMED, Aix Marseille Univ, Marseille, France; 7 Department of Nuclear Medicine, University of Duisburg-Essen and German Cancer Consortium (DKTK) - University Hospital Essen, Essen, Germany; 8 Department of Radiology, Brigham and Women’s Hospital, Boston, MA USA; 9 Department of Radiology, Weill Cornell Medicine, New York, NY USA; 10 Department of Radiology, Mayo Clinic, Rochester, MN USA; 11 Department of Clinical Physiology and Nuclear Medicine, Rigshospitalet, University of Copenhagen, Copenhagen, Denmark; 12 Department of Radiation Oncology, The Ohio State University Wexner Medical Center, Columbus, OH USA; 13 Department of Medicine I, Division of Oncology, Medical University of Vienna, Vienna, Austria; 14 Princess Máxima Centre for Paediatric Oncology, Heidelberglaan 25, 3584 CS Utrecht, Netherlands; 15 Division Imaging & Oncology, University Medical Centre Utrecht, Utrecht, Netherlands; 16 Department of Neurosurgery, Ludwig-Maximilians-University Munich, Munich, Germany; 17 Division of Nuclear Medicine, Department of Biomedical Imaging and Image-Guided Therapy, Medical University of Vienna, Vienna, Austria; 18 Department of Diagnostic and Therapeutic Nuclear Medicine, Clinic Donaustadt, Vienna Health Care Group, Vienna, Austria; 19 Department of Neurology, University Hospital and University of Zurich, Zurich, Switzerland; 20 Department of Neurology, Brain Tumor Center at Erasmus MC Cancer Institute, University Medical Center Rotterdam, Rotterdam, Netherlands; 21 Department of Nuclear Medicine and Nancyclotep Imaging Platform, CHRU Nancy and IADI INSERM UMR 1254, Université de Lorraine, Nancy, France; 22 Department of Neurosurgery, Cleveland Clinic, Cleveland, OH USA; 23 Center for Neuro-Oncology, Dana-Farber Cancer Institute, Boston, MA USA; 24 Institute of Neuroscience and Medicine (INM-3), Research Center Juelich, Juelich, Germany; 25 Department of Neurology, Faculty of Medicine and University Hospital Cologne, Cologne, Germany; 26 Center for Integrated Oncology (CIO), Universities of Aachen, Bonn, Cologne, and Duesseldorf, Cologne, Germany

**Keywords:** Meningioma, PET, Procedure guideline, Somatostatin receptor, DOTATATE, DOTATOC, SiTATE, PRRT, Targeted radionuclide therapy, Theranostics

## Abstract

**Purpose:**

To provide practice guideline/procedure standards for diagnostics and therapy (theranostics) of meningiomas using radiolabeled somatostatin receptor (SSTR) ligands.

**Methods:**

This joint practice guideline/procedure standard was collaboratively developed by the European Association of Nuclear Medicine (EANM), the Society of Nuclear Medicine and Molecular Imaging (SNMMI), the European Association of Neurooncology (EANO), and the PET task force of the Response Assessment in Neurooncology Working Group (PET/RANO).

**Results:**

Positron emission tomography (PET) using somatostatin receptor (SSTR) ligands can detect meningioma tissue with high sensitivity and specificity and may provide clinically relevant information beyond that obtained from structural magnetic resonance imaging (MRI) or computed tomography (CT) imaging alone. SSTR-directed PET imaging can be particularly useful for differential diagnosis, delineation of meningioma extent, detection of osseous involvement, and the differentiation between posttherapeutic scar tissue and tumour recurrence. Moreover, SSTR-peptide receptor radionuclide therapy (PRRT) is an emerging investigational treatment approach for meningioma.

**Conclusion:**

These practice guidelines will define procedure standards for the application of PET imaging in patients with meningiomas and related SSTR-targeted PRRTs in routine practice and clinical trials and will help to harmonize data acquisition and interpretation across centers, facilitate comparability of studies, and to collect larger databases. The current document provides additional information to the evidence-based recommendations from the PET/RANO Working Group regarding the utilization of PET imaging in meningiomas Galldiks (Neuro Oncol. 2017;19(12):1576–87). The information provided should be considered in the context of local conditions and regulations.

## Introduction

In meningiomas, radiolabeled somatostatin receptor (SSTR) ligands are the radiotracers of choice, since meningiomas are characterized by an overexpression of the SSTR type 2 receptor [1]. The [^68^Ga]Gallium-labeled SSTR ligands DOTA-Tyr3-octreotate (DOTATATE), DOTA-Tyr3-octreotide (DOTATOC), DOTA-1-Nal(3)-octreotide (DOTANOC), and, most recently, [^18^F]Fluorine-labeled [^18^F]SiTATE, a SiFAlin-tagged [Tyr3]-octreotate [2], as well as [^64^Cu]Copper-labeled DOTATATE are the most commonly used radiotracers for positron emission tomography (PET) imaging in the clinical management of meningiomas. Besides PET tracers, SSTR-directed tracers for single photon emission computed tomography (SPECT) imaging such as [^111^In]In-pentetreotide or [^99m^Tc]Tc-EDDA-TRYCINE-HYNIC-TOC are available for centers without PET scanners [3, 4], but are not widely used. SSTR tracers provide an excellent lesion-to-background contrast, which also relates to their low uptake in healthy brain parenchyma. The extraordinarily high tracer uptake of meningioma on SSTR PET results in detection sensitivities superior to contrast-enhanced MRI alone [5, 6].

SSTR PET is particularly valuable in cases of ambiguous MRI as well as for the delineation of meningiomas located in regions where morphologic tumor borders are difficult to assess by MRI, such as at the skull base. Furthermore, SSTR PET can be helpful in patients with suspected optic nerve sheath meningioma, as this entity consistently binds SSTR ligands [7]. Due to the high image contrast on PET, small meningeal lesions that have been previously undetected on MRI can be delineated [8, 9], and the extent of meningioma tissue as well as an osseous involvement can be detected with higher accuracy [10], making SSTR PET particularly useful for treatment planning, e.g., for the definition of target volumes prior to radiotherapy [11, 12]. SSTR PET also enables a more precise differentiation between posttherapeutic scar tissue and tumor recurrence [6, 13].

One of the most significant advances in nuclear medicine is the development of theranostic concepts, where an imaging tracer is used for in-vivo visualization of a tumour-specific target, which is subsequently addressed therapeutically by the same or analogous molecule labeled with a therapeutic α- or β^–^-emitter instead of the diagnostic radionuclide. Theranostics with SSTR as target structure are already successfully used in neuroendocrine tumour patients [14], and are potentially applicable to meningiomas. The most widely used radionuclides for SSTR-directed peptide receptor radionuclide therapy (PRRT) are [^177^Lu]Lutetium and [^90^Y]Yttrium, both β^–^-emitters which deposit their high energy within a short range in tissues (max. 2 mm for [^177^Lu] and 12 mm for [^90^Y]) and therefore allow selective irradiation of the targeted tumour lesions. Initial PRRT studies present encouraging results with favorable outcome in patients with treatment-refractory meningioma [15]. Randomized clinical trials on the efficacy of PRRT in meningiomas are missing, therefore PRRT is not yet approved for meningioma patients by medical agencies such as the United States Food and Drug Administration or the European Medical Agency.

The present guideline/procedure standard will focus on the technical aspects of SSTR-directed PET imaging, as well as procedural aspects of SSTR-targeted treatment of meningiomas.

## Goals

The goals of this practice guideline are:

1. to assist healthcare professionals, particularly nuclear medicine practitioners, in recommending, interpreting, and reporting the results of brain SSTR PET imaging in meningiomas, and
2. to define procedure standards for the application of SSTR PET imaging in meningiomas and SSTR-targeted PRRTs in clinical practice and clinical trials in order to facilitate harmonization of data acquisition and treatment protocols across centers.

## Methods

Due to the lack of studies systematically evaluating the clinical impact of different procedural settings and the resulting low level of evidence, recommendations are based on most commonly applied procedures and expert opinions. With regard to the imaging part, procedure standards have been aligned to the most recent guideline for PET imaging of gliomas [16]. The procedures and recommendations for PRRT of meningioma have been adapted from the joint IAEA, EANM, and SNMMI practical guidance on SSTR-directed PRRT in neuroendocrine tumours [17] as well as the NANETS/SNMMI consensus statement on Patient Selection and Appropriate Use of [^177^Lu]Lu-DOTATATE PRRT [18]. The guidelines were brought to the attention of all other EANM Committees and to the National Societies of Nuclear Medicine, as well as to the EANO Guideline Committee.

## Indications and contraindications

### PET imaging

#### Indications

Typical reasons for utilizing SSTR-directed PET imaging in meningioma comprise, but are not confined to, the following:

At primary diagnosis:

- Differential diagnosis between meningioma and non-meningioma tissue [19–21]
- Assessment of tumoral extent for surgical and radiotherapy planning (including identification of bone involvement) [12, 13, 20, 22–26]
- Detection of multifocal disease or extracranial metastases [6, 8, 9, 26, 27]

Disease monitoring and response assessment:

- Detection of residual tumour tissue after surgery [28, 29]
- Response assessment after radiotherapy [26]
- Patient/lesion selection for eligibility for PRRT and response assessment after PRRT [30, 31]

Diagnosis of tumor recurrence /progression:

- Differentiation of meningioma recurrence / progression from treatment-induced changes / scar [5, 6]

The clinical performance of SSTR-directed PET for each indication is discussed in a recent evidence-based recommendation by the PET/RANO Working Group [32]. SSTR-directed PET offers detection of meningioma with high sensitivity and specificity and should be obtained if tumor extension or the diagnosis of recurrence is unclear (evidence class 3, recommendation level C) [33]. Recently, the National Comprehensive Cancer Network (NCCN) Guidelines CNS panel has also updated recommendations to include SSTR-directed PET imaging for diagnostic work-up for meningiomas (NCCN Guidelines Version 1.2023 Central Nervous System Cancers https://www.nccn.org/Register?ReturnUrl=https://www.nccn.org/professionals/physician_gls/pdf/cns.pdf).

#### Contraindications

Relative:

- Pregnancy
- Breast feeding if not discontinued (for further information see [34])

### Targeted radionuclide therapy

#### Indications

SSTR-directed PRRT in meningioma has been investigated in small and uncontrolled studies, mainly including patients with meningioma progressing after multiple prior lines of treatment with positive expression of SSTR type 2 receptors on SSTR-directed PET imaging with potential clinical benefit [15]. Until prospective and adequately controlled clinical trials become available, PRRT remains an investigational treatment and should be considered if other local therapy options (surgery, radiotherapy) are not applicable anymore. Due to the low risk profile and broad experience in patients with neuroendocrine tumours, PRRT may also be offered to patients on an individual basis according to a multidisciplinary assessment.

#### Contraindications

Contraindications for PRRT in meningioma are adapted from the guideline for PRRT in neuroendocrine tumours [17].

Absolute:

- Pregnancy
- Breast feeding (if not discontinued)
- Severe acute concomitant illnesses
- Severe unmanageable psychiatric disorder

Relative:

- Meningioma with mass effect on the brainstem (the risk of edema following PRRT must be discussed in multidisciplinary neuro-oncology board)
- Compromised renal function (glomerular filtration rate (GFR) inferior to 40 ml/min/1.73 m.^2^)
- Compromised bone marrow (white blood cells < 3,000/µl with absolute neutrophil count < 1,000/µl, platelets < 75,000/µl, red blood cells < 3,000,000/µl)
- Hepatic failure with total bilirubin > 3 times the upper limit of normal or albuminaemia < 30 g/l and INR > 1.5
- Heart failure New York Heart Association (NYHA) grade III or IV

Caveats:

- It is essential to exclude or address renal outflow obstruction, which could lead to hydronephrosis and eventual loss of renal function, before initiating PRRT.
- Prior myelotoxic chemotherapy and extensive external beam irradiation to the bone marrow (such as in the pelvis or spine), particularly within the 8 weeks preceding PRRT, increase the risk of bone marrow toxicity post-PRRT.
- In patients with impending liver or heart failure risks and benefits of PRRT should be considered cautiously.

## Qualification and responsibilities of the personnel

### PET Imaging

#### Physician

PET examinations should be performed by, or under supervision of, a physician specialized in nuclear medicine and certified by accrediting boards. In Europe, the certified nuclear medicine physician who performed the study and signed the report is responsible for the procedure, according to national laws and rules. In the United States, the SNMMI Guideline for General Imaging (Society of Nuclear Medicine, 2010, http://interactive.snm.org/docs/General_Imaging_Version_6.0.pdf) should be considered.

### Technologist

PET examinations should be performed by qualified registered/certified Nuclear Medicine Technologists. Please refer to: Performance Responsibility and Guidelines for Nuclear Medicine Technologists 3.1 and http://www.eanm.org/content-eanm/uploads/2016/11/EANM_2017_TC_Benchmark.pdf for further details. In some jurisdictions, there may be additional qualifications necessary for technologists to operate the CT or MR components.

### Physicist

PET scans should be conducted using PET systems that adhere to national or international quality benchmarks. A certified clinical physicist is responsible for ensuring that PET systems meet these standards. Furthermore, in certain countries, it is mandatory to have a board-certified medical physicist accessible to advise or assist the personnel responsible for operating imaging systems and addressing any dysfunction. Additionally, scans should adhere to national or international dosimetry and radiation safety protocols, ensuring safety for both patients and staff.

### Targeted radionuclide therapy

PRRT as a therapy for meningioma patients is considered an investigational treatment, requiring adherence to national legislation, local requirements, and ethical standards governing human studies. Facility specifications vary based on national regulations regarding the therapeutic use of radioactive agents, with some countries mandating inpatient therapy. Facilities must possess suitable personnel, radiation safety equipment, and protocols for waste management and handling accidental contamination. The administration of [^90^Y]- or [^177^Lu]-labeled SSTR ligands should be conducted by trained medical staff with nursing support and a medical physics expert. Treating physicians should possess a general understanding of the disease's pathophysiology and natural progression, familiarity with alternative therapies, and the ability to collaborate closely with other specialists. Referring physicians utilizing unsealed radionuclide sources must comply with relevant national legislation and local regulations. The decision to recommend PRRT as an experimental anti-tumor therapy should be made within a multidisciplinary neuro-oncology board involving all relevant specialists. Whenever possible, meningioma patients should be enrolled in clinical trials for PRRT treatment.

## Procedure and specifications

### PET imaging

#### Prerequisites and patient preparation

- Adequate clinical details about the patient and a clearly defined clinical query must be established to justify PET imaging.
- Information regarding the patient's ability to cooperate during the examination and the presence of a caregiver for assistance can be beneficial.
- Documenting the patient's prior therapeutic history, including surgeries, radiotherapy, and chemotherapy, which may impact radiopharmaceutical distribution and toxicity, is essential. Additionally, history of non-radioactive somatostatin analog usage, including type and date of last injection, if applicable, should be noted.
- Obtaining relevant imaging study results, surgical resections, and biopsy reports is recommended.
- Pre-screen all patients for MRI contraindications using a standardized checklist, particularly if SSTR imaging is conducted using PET/MRI.
- Record height and body weight which are needed for Standard Uptake Value (SUV) calculations.
- If available, recent anatomical imaging with MRI (T1-weighted sequence pre- and post-contrast, FLAIR, and/or T2-weighted sequence) should be obtained for image fusion.
- Ensure that the patient is informed by a physician about the procedure to ensure optimal compliance. Document patient information and consent as per local regulations.
- Patients should be capable of remaining still for at least 10 to 15 min during the scan.
- If sedation is necessary, administer it according to local standard operating procedures, typically starting 20–60 min before the examination.
- Advise patients to empty their bladder before scanning to enhance comfort and reduce absorbed dose to the bladder.
- In the case of pregnancy, a clinical decision should weigh the benefits against potential risks.
- If PET imaging will be performed using PET/MRI, confirm MRI contraindications and take appropriate measures.
- Remove all metal objects from the patient and provide cotton clothing to prevent artifacts during MRI.
- Obtain information about any implants or devices, including type, location, and material, and verify their safety for MRI scanning. For "MRI conditional" implants, document all conditions for safe scanning.
- Encourage patients to stay hydrated and empty their bladder frequently before the examination.

#### Radiopharmaceuticals

The most commonly used PET tracers for SSTR imaging in meningioma are:

- [^68^Ga]Ga-DOTATOC
- [^68^Ga]Ga-DOTATATE
- [^64^Cu]Cu-DOTATATE
- [^64^Cu]Cu-DOTATOC
- [^68^ Ga]Ga-DOTANOC
- [^18^F]SiTATE

The above mentioned [^68^Ga]-labeled tracers are in detail described in the “defintions” section of the practice guideline on PRRT in neuroendocrine tumours [17]. In the US, only [^68^Ga]Ga-DOTATATE as well as [^64^Cu]Cu-DOTATATE are currently availably in routine clinical setting. [^18^F]SiTATE is a novel PET tracer targeting the SSTR receptor subtype 2 [2] with high tumor-to-background contrast in neuroendocrine tumours [35] and meningioma [9, 36]. Due to the labeling with [^18^F] and consecutively longer tracer half-life of 110 min, [^18^F]SiTATE may have logistic advantages over the [^68^ Ga]-labeled tracers as more patients can be scanned with one production and the tracer can be delivered to distant PET centers. The relative cost-effectiveness of PET imaging with the various SSTR ligands and modalities in specific clinical settings and regulatory environments is to be established [37].

##### Preparation of the radiopharmaceuticals

The current regulatory framework for preparation of radiopharmaceuticals is not harmonized throughout the world, however, it should be performed by qualified personnel, and comply with a strict quality control and local regulatory requirements [38, 39].

##### Administered activity

The injected activity should be adapted to national regulations and the used PET technology. The most commonly used activities for SSTR PET in adults are currently as follows:

- [^68^ Ga]Ga-DOTATOC: 100–200 MBq
- [^68^ Ga]Ga-DOTATATE: 150–200 MBq
- [^68^ Ga]Ga-DOTANOC: 150–200 MBq
- [^64^Cu]Cu-DOTATATE: 150–250 MBq
- [^64^Cu]Cu-DOTATOC: 150–250 MBq
- [^18^F]SiTATE: 150–250 MBq

Injected activity for children should be reduced and adapted to the weight in compliance with the EANM Paediatric Dosage Card or 2016 Update of the North American Consensus Guidelines for Pediatric Administered Radiopharmaceutical Activities [40].

#### PET acquisition protocols

Generally, image acquisition of the head is sufficient. Additional whole-body (skull base to thigh) acquisition can be considered in individual patients (e.g. in case of symptoms indicating potential metastatic spread, to exclude/detect metastases or spinal meningioma) [41]. During the entire investigation, continuous visual monitoring of the patient is necessary. Monitoring is particularly important in patients with tumour-associated seizures.

##### Positioning

The patient scan should be performed while positioning the patient in a dedicated head holder with arms positioned along the body. If a whole-body scan is planned and the acquisition is performed in two separate steps (first head, then rest of the body), the rest of the body should be scanned with arms up to reduce artefacts in the area of interest. The entire brain should be in the field of view, including the entire cerebellum. Avoid extreme neck extension or flexion.

##### Head stability

The patient should be informed immediately before PET acquisition to avoid head movements during all parts of the investigation. Head stability can be obtained by comfortably positioning the patient in the head holder and securing the head as completely as possible. Tape, padding or other flexible head restraints including termoplastic mould and vacuum mattress for children may be employed and are often helpful, especially for radiotherapy planning purposes.

##### PET imaging sequence

The preferred sequence of PET imaging is:

- CT scout topogram for PET/CT to setup field of view
- For attenuation correction: Low- or high-dose CT scan, MRI attenuation correction scan or transmission scan.
- Static single Field of View (FOV) PET acquisition of the head at 60 min p.i.; emission image acquisition time varies depending on the administered activity, patient body weight, and the sensitivity of the PET device (typically around 10-20 min); in case of additional whole-body PET/CT followed by acquisition of the skull base to thigh (typically between 1-5 min per bed position). The PET/CT of the body can be acquired either within one acquisition process or in two separate steps (first head, then second PET/CT acquisition of the body) depending on the scanner system.
- Dynamic PET acquisition and generation of parametric images may provide additionale information, but are not part of the clinical routine and remain investigational [42].

##### Attenuation correction

Image acquisition should be conducted in 3D data acquisition mode, and attenuation correction should be performed using (low-dose) CT, MRI attenuation correction, or a 511 keV-transmission scan. If a 511 keV-transmission scan is utilized, it should be obtained prior to tracer injection. CT parameters should be selected to minimize patient dose while fulfilling the intended purpose.

Regarding attenuation correction in PET/MRI:

- MRI accuracy and artifact-free images are crucial for precise PET quantification, especially in meningioma, which often have peri- or intraosseous locations, as errors in MRI attenuation correction estimation are most significant in bone assessment [43].
- Utilize the latest MRI attenuation correction software, incorporating ultrashort echo time (UTE), zero TE (ZTE) sequences, or bone models for improved bone detection in brain PET/MRI studies, where available [44, 45].
- Recognize that MRI attenuation correction software may vary among vendors and undergo continuous updates with potentially undocumented performance changes.
- Several attenuation correction strategies for PET/MRI exist, some of which may introduce systematic differences in activity distribution and calculated semiquantitative metrics, necessitating careful consideration during PET image interpretation [46–48].
- MRI attenuation correction images must routinely be checked for artefacts, consistency and plausibility during PET/MRI reading. Artefacts in MRI attenuation correction have a direct effect on PET quantification in brain PET/MRI. Typical artefacts are missegmentation of brain / fat / bone tissue, metal artefacts due to dental prostheses and due to metallic implants such as coils, stents, surgical clips, titanium calvarial implant/mesh etc. [46, 49, 50]. Artefacts may show as signal voids, exceeding the true dimensions of metal inclusions. Thus, artefacts are mostly well detectable in MRI attenuation correction indicating regions of potentially inaccurate PET quantification [51].
- When applicable, employ time-of-flight (TOF) PET detection to mitigate the influence of metal artifacts in brain PET/MRI scans [52].
- Utilize radiofrequency head coils specifically designated for combined PET/MRI applications. The use of standard radiofrequency head coils intended solely for MRI can result in exclusion from PET/MRI attenuation correction, potentially resulting in inaccuracies in PET quantification and the presence of artifacts in PET images.
- As the effects of MRI attenuation correction on meningioma delineation and quantification is unknown, it is recommended not to use MRI attenuation correction in radiation treatment planning. However, time-of-flight-derived attenuation maps and subsequent attenuation correction are an interesting development for TOF PET/MRI systems [53].

##### PET comparability

For longitudinal studies, whenever feasible, patients should be scanned on the same system using consistent procedures to minimize variations due to differences in imaging technology or methodology. To maintain PET comparability, employ a standardized protocol for image acquisition and clinical interpretation. Refer to the sections on "PET image reconstruction" and "Documentation and reporting" for more detailed information.

#### PET image reconstruction

In image reconstruction, all corrections necessary for quantitative assessment are imperative, including attenuation, scatter, random, dead time, and decay corrections, along with detector sensitivity normalization. While TOF acquisitions and reconstructions are permissible, their advantages for brain imaging are not yet fully explored. Iterative reconstruction is currently the standard in the field and should be utilized. Incorporating resolution modeling during reconstruction, known as point spread function (PSF) reconstructions, may enhance the delineation of meningioma borders and improve detectability, albeit with a potential for Gibbs artifacts [54] and quantitative errors dependent on tumor size and PET/CT scanner model.

In order to harmonize PET image quality, especially for multi-center settings, EARL requirements for IQ recovery have been issued [55]. Typically, employing a higher resolution reconstruction can aid in visual interpretation and tumor delineation. If a particular PET system permits the use of multiple reconstruction methods, a dedicated high-resolution brain reconstruction protocol may be utilized.

#### Interpretation / Quantification

##### General image display

PET images should contain pixels with a minimum depth of 16 bits to ensure a sufficient range of values, and suitable image scaling techniques should be utilized for display purposes. Incorporating a color scale is permissible. Displaying PET images in the transaxial orientation is recommended, along with correlation with morphological images in the coronal and sagittal planes. Internal landmarks can aid in reorientation to achieve a standardized image display, with procedures typically based on the intercommissural line being commonly employed [56].

##### Image analysis

Calculation of the standardized uptake value (SUV) is essential and can be carried out by dividing the tissue's radioactivity concentration (kBq/ml) by the injected radioactivity (MBq) per body weight (kg), body surface area (m^2^), or lean body mass (kg), depending on the most suitable distribution volume for each tracer.

Standard summation images are utilized for clinical interpretation and are assessed alongside (contrast-enhanced) CT and/or MRI scans.

During initial visual analysis, a subjective qualitative assessment may be conducted, classifying the lesion of interest as either *positive*, characterized by visually intense and compact tracer uptake, or *negative*, where little or no uptake is observed. SUV_max_ values are assessed in the lesion(s) of interest. Systematic data that inform specific recommendations on optimal reference regions and thresholds defining PET-positivity are not available. In different centers, various methods including the assessment of SUV ratios (e.g. comparison to the superior sagittal sinus (SUVR_SSS_) [42, 57, 58], contralateral brain meninges [59], liver in case of wholebody scanning [60, 61]) or histologically verified SUV thresholds (2.3 for [^68^Ga]Ga-DOTATATE [6]) are being utilized. In contrast to brain tumor imaging with radiolabelled amino acids, the healthy brain parenchyma in the contralateral hemisphere or in the centrum semi-ovale does not serve as reference region. Further research including structured evaluation of analytical performance of determination of PET-positivity including interinstitutional reproducibility and its clinical performance (correlation with response to PRRT) is encouraged.

##### Cut-off thresholds for definition of biological tumor volume

Methods for tumor delineation on SSTR PET are still under discussion. So far, only one study has systematically compared uptake values on SSTR PET to histological results based on neuronavigated tissue sampling and suggested a SUV of 2.3 on [^68^Ga]Ga-DOTATATE PET as optimal threshold for the differentiation between meningioma tissue and non-neoplastic tissue [6]. A recent study suggested a 1.7 fold meninges SUV_peak_ as threshold for semiautomatic delineation of tumor volume [59]. SSTR PET-based tumor volume definition for radiotherapy planning has been investigated in few retrospective and small studies which indicated potentially increased accuracy and decreased interobserver variability compared to MRI [13, 62]. Overall, further studies are warranted to assess the optimal method for standardized tumor segmentation and threshold definitions.

##### Interpretation of PET data

At primary diagnosis:

- Negative scan: Low uptake (i. e. with a SUV < 2.3 or SUVR_SSS_ of < 3) excludes a meningioma with high probability. However, rare meningioma cases may present with low uptake on SSTR PET [63]. So far, no clear correlation of tumour grade or meningioma subtype with lack of SSTR PET positivity has been established.
- Positive scan: Elevated uptake, which exhibits a high positive predictive value for a meningioma [6]. A reliable differentiation of WHO grades based on uptake intensity is not possible. Importantly, also other neoplastic as well as rare cases of non-neoplastic lesions can show increased uptake, e.g. brain metastases, gliomas, and primary CNS lymphoma [21, 64–66], however, in most of the cases with lower overall uptake intensity.

For treatment planning:

- Areas with meningioma-suspicious uptake may be used to support delineation of the SSTR-positive tumor tissue and osseous involvement for surgery and radiotherapy planification, and should be interpreted in conjuction with fused MRI and CT scans [13].

At suspected tumor recurrence:

- Increased uptake in the follow-up of previously treated meningioma has high accuracy in differentiating recurrent disease from treatment-related changes (e.g. radionecrosis / scar tissue) [6].

For response assessment:

- Response assessment to therapeutics interventions in meningiomas is currently performed using MRI-based criteria such as modified Macdonald criteria or RANO criteria [67, 68]. A framework for PET-based response assessment as published for diffuse glioma [69] is currently not available. The increasing intensity of tracer uptake throughout various therapies does not necessarily signify therapy failure, whereas diminishing uptake may indicate a positive response [11]. In a recent interim analysis of an ongoing phase II trial investigating [^177^Lu]Lu-DOTATATE treatment for progressive meningioma, > 25% reduction in [^68^Ga]Ga-DOTATATE uptake was observed in five meningiomas and two patients. In one lesion, this corresponded to > 50% reduction in bidirectional tumor measurements on MRI [70].

##### Physiological tracer distribution

- High physiologic uptake in pituitary gland (which may cary depending on patient’s age and sex [71])
- Slight uptake in vascular structures, choroid plexus, salivary glands

##### Known pitfalls

- Active inflammatory lesions may present increase uptake (e.g. granulomatous inflammation and neurosarcoidosis [66]).
- Other neoplastic lesions (e.g. brain metastases [64] and glioma [72]) may also present slightly increased uptake.
- Uptake may be decreased or absent in rare cases of meningioma [63]

### Targeted radionuclide therapy

This section presents procedures for PRRT in meningiomas. Most procedures are similar to PRRT applied in neuroendocrine tumors and have therefore been adapted from the joint practical guidance of the International Atomic Energy Agency (IAEA), EANM and SNMMI on peptide receptor radionuclide therapy in neuroendocrine tumors [17] as well as the NANETS/SNMMI Procedure Standard for SSTR-based PRRT with [^177^Lu]Lu-DOTATATE [4] and Consensus Statement on Patient Selection and Appropriate Use in neuroendocrine tumors [18]. The current guideline is nonetheless adjusted to meningiomas.

#### Request

The patient should have received a complete comprehensive briefing about the PRRT, its expected results and potential side effects during a consultation with a physician and has given his/her consent for this treatment. Patient information and consent should be documented in the patient files. A booklet summarizing the procedure of PRRT, the potential side effects, the rules for radiation protection to follow may be provided to the patient during this consultation.

The following criteria should be checked and fulfilled for general eligibility before planning the PRRT:

- Meningioma with positive somatostatin receptor expression on SSTR PET imaging within the last 2 months.
- Karnofsky performance status above 60% or ECOG 0–2.
- Brain MRI within the last 2 weeks prior to PRRT as baseline for further disease monitoring and response assessment.
- No contraindications to PRRT as mentioned in the dedicated paragraph above.

#### Patient preparation and precautions

Hospitalization after PRRT depends on national legislations and institutional regulations.

##### Prior to PRRT

Physicians responsible for PRRT must check the recent laboratory tests including complete blood count with platelets, renal function (with GFR calculation), blood ionogram (potassium), liver function (alanine transaminase (ALT), aspartate transaminase (AST), alkaline phosphatase (ALP), gamma-glutamyl transferase (GGT), serum bilirubin, albumin) before the initiation of PRRT. Pregnancy must be excluded in women of childbearing age (e.g. by urinary pregnancy test or β-HCG blood levels).

Before initiating PRRT, it's advisable to discontinue somatostatin analogs, as they could potentially disrupt receptor targeting. The length of interruption varies depending on the half-life of the analog used. Generally, withdrawal periods of 4–6 weeks for long-acting release formulations and at least 24 h for short-acting formulations are recommended as good clinical practice. If not considered necessary, corticosteroids should be stopped or at least tapered to the smallest required dose as they may decrease the efficacy of PRRT by downregulating the SSTR-2 expression [73]. However, if edema before or during PRRT requires corticosteroids, these should be given at the effective dose.

The day before the treatment, the patient should be hydrated adequately (at least 500 ml of water 12 h before the beginning of the treatment).

##### On the day of PRRT

On the day of the administration of PRRT, large meals are not recommended due to the possibility of nausea related to amino acid infusion as a common side effect.

For i.v. administration, two venous accesses are recommended, one for the radiopharmaceutical administration, the other for the amino-acid infusion. A double chamber catheter port is an alternative option.

To mitigate nephrotoxicity and minimize excessive kidney retention of radiopharmaceuticals, positively charged amino acids like L-lysine and/or L-arginine are co-infused. This serves to competitively inhibit the proximal tubular reabsorption of the radiopharmaceutical. The typical infusion consists of lysine (25 g) and arginine (25 g) in large volumes of 0.9% NaCl, usually 2 L, unless the patient has cardiac insufficiency. This infusion is recommended to be started 30 min to 1 h before the administration of PRRT and maintained over 4 to 6 h by slow intravenous administration with a flow rate of 250 ml to 500 ml/hour. Other proposed amino acid protective schemes are possible, but have logistic disadvantages (e.g., 3-day protocol) or reported allergic reactions when applied with Gelofusine [4, 17]. For individuals with significant cardiac insufficiency, it's essential to prevent volume overload, which could precipitate acute cardiac insufficiency and decompensation. Consequently, formulations containing reduced amounts of amino acids and thus lower volumes should be selected. For instance, 25 g of lysine or arginine could be diluted in a maximum of 1 L of normal saline.

Anti-emetic agents (metoclopramide or ondansetron) can be prescribed prior to amino acid infusion and may be repeated if needed.

#### Radiopharmaceuticals

The most commonly used SSTR2-directed therapeutic radiopharmaceuticals are

- [^177^Lu]Lu-DOTATATE/DOTATOC
- [^90^Y]Y-DOTATATE/DOTATOC

##### Administered activity in adults

Irrespectively of the radiopharmaceutical used, the treatment regimen is commonly based on standard doses of 7.4 GBq for [^177^Lu]-labeled radioligands and 3.7 GBq for [^90^Y]-labeled radioligands [4, 17]. In case of renal failure, it is preferable to administer [^177^Lu]Lu-DOTATATE, which is associated with lower renal toxicity [60]. The injected activity can be reduced (e.g., 3.7 GBq for [^177^Lu]Lu-DOTATATE) if reversible clinical-biological side effects are observed after previous treatment, similar to PRRT in NETs [4, 17].

#### Administration of PRRT

As detailed in the patient preparation paragraph, each administration of SSTR-ligands PRRT must be preceded/accompanied by amino acid infusion.

The radiopharmaceutical needs to be mixed with saline, with the final volume ranging between 10 and 100 ml, contingent on the infusion system utilized. It should be administered through a peripheral intravenous access over a duration of 10 to 30 min, depending on the infusion system employed. The radiopharmaceutical is dispensed from a vial under positive pressure and can be administered via gravity, infusion pump, or automated syringe pump injector methods. A physician must be present nearby during the administration of the radiopharmaceutical.

Intraarterial administration instead of standard intravenous administration of PRRT has been reported to increase tracer accumulation in a case series [74] and may therefore increase treatment efficacy. However, an intraarterial injection of radioligands is associated with challenging logistics, requires an interventional (neuro-)radiologist as well as the license for handling radioactive substances outside of the nuclear medicine department. Future prospective studies investigating intra-arterial PRRT in meningioma are necessary to assess potential improvements in treatment efficacy.

The patient must remain under surveillance after the treatment administration to take into account potential side effects: nausea, vomiting, rarely epileptic seizures or signs of intracranial hypertension. Symptomatic therapy can be given, if necessary, after the PRRT administration (e.g., antiemetics, anticonvulsive drugs, corticosteroids).

Radiation protection rules must be followed according to local regulations. Patients should stay adequately hydrated (at least 1 l of water per day), wash their hands after urination, collect all waste that could potentially be contaminated by irradiation, and the need to keep all waste at distance for sufficient radiation decay. Incontinent patients should be catheterized prior to PRRT and the catheter should be kept for up to 2 days thereafter, if necessary. Urine bags should be regularly emptied by personnel wearing appropriate protective gear. Additionally, patients should be instructed to refrain from close contact with infants and pregnant women during the initial days following treatment, in accordance with national regulations, at least 3 days after a treatment with [^90^Y] and 7 days after a treatment with [^177^Lu].

#### Targeted radionuclide therapy regimens

PRRT regimens in patients with meningiomas are usually based on 4 treatment cycles spaced 8 ± 2 weeks apart in analogy to the treatment regimens in patients with neuroendocrine tumors. Prospective trials are needed to explore safety and efficacy of alternative dosing regimens. If PRRT cannot be administered when the patient is due for their next cycle, it can be postponed up to 16 weeks without any changes to the remainder of the treatment regimen.

Combination therapies with concurrent therapies may be beneficial [75, 76], but are not yet systematically investigated.

#### Dosimetry

Assessment of radiation dosimetry in normal organs and meningioma lesions offers valuable insights into the absorbed doses of PRRT and may aid in treatment optimization. Patient-specific dosimetry, when feasible, can provide crucial information for evaluating organ-specific radiation absorbed doses and assessing the risk of delayed organ toxicity, particularly in patients with known risk factors for kidneys and bone marrow.

Reported absorbed radiation dose estimates following PRRT using [^90^Y]Y-DOTATOC range from 0.03 ± 0.01 to 0.17 ± 0.02 Gy/GBq for bone marrow, 1.71 ± 0.89 to 2.84 ± 0.64 Gy/GBq for kidneys, and from 0.27 to 1.5 ± 1.2 Gy/GBq for the liver. Corresponding dose estimates for [^177^Lu]Lu-DOTATATE range from 0.02 ± 0.03 to 0.07 ± 0.01 Gy/GBq for bone marrow, 0.62 to 0.90 ± 0.30 Gy/GBq for kidneys, and from 0.13 to 0.21 ± 0.08 Gy/GBq for the liver.

Various dosimetry methods, both practical and sophisticated, can be utilized depending on the ultimate objective and resource availability. Input data include information from blood, urine, and whole-body scans conducted adequately up to at least 3 days post-PRRT. Planar images are useful for deriving biokinetics over time, while SPECT(/CT) images offer insights into organ-specific three-dimensional activity distribution. The MIRD scheme provides reference techniques for internal dosimetry.

For [^177^Lu]Lu-DOTATATE, gamma photon emission enables both imaging and dosimetry of the same compound, allowing dosimetry to be conducted during the initial courses of therapy following the injection of [^177^Lu]Lu-DOTATATE. The most common scheme for [^177^Lu]Lu-DOTATATE for dosimetry involves posttherapeutic imaging with at least 3 time points, e.g., between 1 and 4 h p.i., 24 h p.i., 48 h p.i., 72 h p.i., at day 7 if possible [77]. The imaging should be performed preferably via SPECT(/CT). In the case of [^90^Y]Y-DOTATOC, the absence of γ emission from [^90^Y] poses challenges for direct dosimetry. Quantifying Bremsstrahlung images is quite challenging, often necessitating the implementation of intricate corrections.

#### Side effects

Adverse events after PRRT have been systematically assessed in patients with neuroendocrine tumours [14]. Common Terminology Criteria for Adverse Events (CTCAE) grade 3 and 4 adverse events occur in approximately 40% of patients, but are generally clinically well manageable and most commonly include nausea (4%), vomiting (7%) and lymphopenia (9%) [14]. Reported experience in retrospective and small meningioma studies is in line with these findings.

##### Acute side effects

Adverse effects of PRRT are typically mild, and precautions are taken as needed. Nausea, headache, and occasionally vomiting may occur in the majority of patients due to metabolic acidosis induced by amino acid co-administration. Special attention should be given to prevent potential electrolyte imbalances (such as hyperkalemia and hypernatremia) and subsequent metabolic acidosis, which can result in mild nausea and vomiting. Hydrating the patient with normal saline and possibly repeating antiemetic administrations can help manage these side effects. In the context of meningiomas, patients may experience acute post-treatment epileptic seizures or signs of intracranial hypertension, for which antiepileptic drugs or corticosteroids should be prescribed accordingly.

Regarding radiotracer extravasation leading to potential soft tissue damage little is known [78]. In case of radiotracer extravasation, discontinuation of the radiotracer infusion is recommended. The application of warm compresses to limit extravasation of the radiotracer for 20 min with repetition at 6, 18 and 24 h after injection can be considered [79]. Raising the affected limb and stimulating lymphatic drainage of this small radiopharmaceutical may reduce local accumulation overnight. Notify the person competent in radiation protection for a dosimetry study.

##### Chronic side effects

In the event of a significant laboratory abnormality, or if clinical or laboratory evidence of toxicity occurs, additional specimens for repeat or additional analyses should be collected [14].

In general, any CTCAE grade 3 and 4 toxicity (except lymphocytopenia) should postpone the course until complete resolution or return to initial value. For the following course, the fixed standard dose should be reduced (half-dose), and if no abnormality is observed with a reduced dose, fixed standard dose can be prescribed for the next courses. A recurrence of toxicity leads to the definitive termination of PRRT [14]. Personalised treatment taking into account the prognosis and need to stabilize the disease or induce a response can justify the administration of higher activities, preferentially in the context of clinical trials.

The potential influence of tumour volume, localization and prior or concurrent cumulative irradiation on the development of adverse events (e.g. symptomatic edema, radionecrosis) is currently unknown. Although baseline evaluation and monitoring of pituitary hormone levels was not mandated in the registration trial of PRRT in neuroendocrine tumours [14], monitoring of hormone levels should be considered in individual patients (e.g. known pituitary dysfunction, meningiomas in close proximity to the pituitary gland).

Secondary myelodysplastic syndromes and acute myeloid leukaemia have been reported in less than 3% of cases, and long-term results of the NETTER-1 trial indiciated that there was no apparent long-term nephrotoxicity with [^177^Lu]Lu-DOTATATE treatment [80].

#### Follow-up

##### Clinical monitoring

The evaluation of renal function holds significant importance, given that the kidney, along with the bone marrow, is a crucial organ in PRRT. Therefore, serum creatinine levels and the determination of glomerular filtration rate (GFR) should be assessed before each cycle of PRRT and during follow-up. For patients with pre-existing risk factors for delayed renal toxicity—such as longstanding hypertension, diabetes mellitus, single kidney, previous renal injury, or exposure to nephrotoxic chemotherapy—additional scintigraphic methods to assess renal function may be considered if available. These methods may include measuring [^99m^Tc]Tc-MAG_3_ clearance or GFR using [^99m^Tc]Tc-DTPA or [^51^Cr]Cr-EDTA.

Regular bloodwork including complete blood cell count, renal and liver function tests should be performed at 2–4 weeks intervals during PRRT cycles, every 8–12 weeks for the first 12 months after the last PRRT cycle, and can be followed on an annual or semiannual basis if clinically indicated) should be prescribed, involving complete blood cell count. Renal and liver function tests should be performed prior to a subsequent cycle of PRRT. A special attention must be paid to the risk of lymphopenia which increases with PRRT, especially with patients receiving additional immunosuppressive medications and who underwent previous cytotoxic chemotherapy.

##### Imaging

An imaging follow-up 1 month after the second course of treatment including a brain MRI and a SSTR PET imaging is necessary to assess response and to exclude progression. At the end of the four cycles of treatment, a final imaging control including a brain MRI and a SSTR PET imaging should be planned 2 months after the end of the treatment.

##### Retreatment options

The decision to re-administer PRRT to a patient should only be made within the context of a multidisciplinary tumor board. For patients who have previously responded to PRRT, retreatment may be contemplated in cases of documented disease progression, with consideration given to the cumulative radiation dose received by the kidneys and bone marrow. The eligibility criteria for this subsequent PRRT course will align with those applied during the initial radiopharmaceutical treatment cycle. As a general guideline, meningioma patients should ideally undergo PRRT within clinical trials, when feasible.

## Documentation and reporting

### PET imaging

The description of findings in brain tumor imaging should typically adhere to previously published guidelines for FDG imaging in oncology, as well as for brain tumor imaging using radiolabeled amino acids and FDG. This includes considerations regarding general aspects of reporting such as thoroughness [16, 81].

The content of the report plays a crucial role in patient management and serves as a legal document. It is advisable to furnish a structured report with succinct concluding statements aimed at addressing the specific clinical question(s) posed, whenever feasible.

Reports should encompass the following general structure:

- General information:
- Name of the patient and other identifiers, such as birthdate
- Name of the referring physician
- Type and date of examination
- Details regarding the radiopharmaceutical, including the route of administration and the administered activity amount
- Patient history, with a focus on diagnosis, tumor-related treatments, and the clinical question prompting the study request
- Documentation of patient information and consent

Body of the report:

- Procedure description:
- Imaging procedure performed (e.g. PET, PET/CT, PET/MRI), contrast media and interval between PET tracer injection and image acquisition
- If sedation is performed, describe type and time of medication in relation to the tracer injection
- Data quality:
- Abnormal tracer biodistribution
- CT-related or MRI attenuation related artifacts e.g. from metallic implants
- Any observed events that could potentially affect interpretation, such as head movements or seizure activity
- Comparative data:
- Registration and comparison of PET/CT images with MRI
- Comparison of PET/CT images with previous MRI and PET/CT scans to assess disease progression
- The type and date of comparative data should be specified before describing imaging findings
- Description of findings:
- It should be stated if radiotracer uptake is normal or abnormal
- In case of abnormal findings, an anatomically correct description of location, extent, and intensity of pathological tracer accumulation relative to normal tissue uptake should be described. 
-  Characteristics of uptake should include:
  - Patterns of uptake (e.g., focal, diffuse, inhomogeneous)
  - Intensity of uptake (visually slight, moderate, or strong, including SUV_max_)
  - Correlation with morphological imaging (e.g., abnormalities on CT/MRI)
- Semiquantitative parameters:
  - SUV_max_ for each lesion of interest; reporting of the biological (SSTR-positive) tumor volume is optional.
- Clinically relevant incidental findings, like extracerebral metastases, should be reported.
- Comparison to previously performed PET studies for treatment response or tumor progression diagnosis is important.
- Limitations:
  - Factors affecting data quality or diagnostic accuracy, should be mentioned when appropriate.

Interpretation:

- Interpretation should address the clinical question, integrating medical history, comparative imaging, and any limitations. A precise diagnosis should be provided whenever possible, with recommendations for additional or follow-up scans as appropriate.

### Targeted radionuclide therapy (after each PRRT cycle)

General information:

- Name of the patient and other identifiers, such as birthdate
- Name of the referring physician
- Type and date of treatment: hospitalization or outpatient
- Details regarding the radiopharmaceutical, including the mode of administration and the administered activity amount
- Patient history, with a focus on diagnosis and tumor-related therapy
- Known allergies and hypersensitivities
- Documentation of patient information and consent

Body of the report:

- Number of the course of PRRT
- Details of the clinical reports before PRRT
- Detailed procedures: antiemetic, amino-acid injection, other symptomatic therapy
- Course of PRRT, potential acute side effects observed and whether they have been managed
- Dosimetry (if performed) and topography of radiotracer uptake observed if SPECT imaging have been performed following the course of the treatment
- Reminder of radiation protection rules given to the patient
- Prescriptions for medication as well as biological assessment / controls of laboratory parameters to be carried out
- Reminder of dates of following medical consultations and if necessary following brain MRI or SSTR PET imaging scheduled.

## Equipment Specifications

### System specifications

It is advised to utilize state-of-the-art 3D PET/CT or PET/MRI systems. These systems should be capable of acquiring (low-dose) CT images or MRI sequences suitable for attenuation and scatter correction of PET emission data. Alternatively, dedicated brain PET-only systems may be employed, provided they are equipped with transmission scan sources of adequate strength, as recommended by the vendor, to ensure high-quality transmission scans and, consequently, accurate PET emission data attenuation correction. PET(/CT) systems should have a minimum axial field of view of 15 cm to adequately cover the entire brain, including the cerebellum and brain stem.

### PET acquisition

The system should possess the capability to acquire PET emission data in 3D mode. Data reconstruction can occur either online or offline (i.e., retrospectively), in single or multiple frames. Additionally, PET images may be reconstructed with or without attenuation correction. While non-attenuation corrected PET images are not primarily used for interpretation, they can aid in identifying attenuation artifacts in the attenuation-corrected PET images. The system should encompass all functionalities and methods necessary for quantitative brain PET imaging and reconstruction, including but not limited to online randoms correction, scatter correction, attenuation correction, dead time correction, decay and abundance correction, and normalization (i.e., correction for detector sensitivities).

### SPECT acquisition

To conduct dosimetry with ^177^Lu/^90^Y, SPECT/CT systems must be capable of acquiring at least whole-body planar images with quantification. Acquiring 3D whole-body SPECT/CT images may enhance dosimetry accuracy. The camera system should possess all functionalities and methods necessary for quantitative SPECT imaging and reconstruction, including but not limited to scatter correction, attenuation correction, and calibration.

## Quality control and improvement

Quality and quantification of PET images is affected by various factors [82]. To ensure superior image quality, quantitative accuracy, and image consistency, especially crucial for PET image comparability in multicenter studies, the correct performance of PET systems must be regularly checked by several QC experiments. As QC experiments have in detail been described presviously, we here refer to the corresponding paragraph in other guidelines [16].

## Conclusion

The clinical utilization of SSTR-directed PET/CT imaging and PRRT among patients with meningioma has shown a consistent rise in Europe and the US. To ensure effective and suitable implementation, standardized protocols and procedures are imperative. This document aims to offer guidance on conducting and interpreting SSTR-directed PET imaging and therapy for meningiomas, aiming to complement recent clinical guidelines and assist in formulating clinical trial protocols [32]. As systematic studies evaluating the clinical impact of different procedural settings are still lacking, recommendations are mainly based on expert opinions. In order to bring SSTR-directed PET/CT imaging as well as SSTR-directed radionuclide therapy of meningiomas into daily clinical practice and to increase the evidence level of this highly promising theranostic approach, adequately designed and controlled prospective multicenter trials are strongly encouraged.

## Liability statement

This guideline summarizes the views of the EANM Neuroimaging and Oncology & Theranostics Committees, of the EANO Guideline Committee, the PET taskforce of the RANO group and the SNMMI. It reflects recommendations for which the EANM cannot be held responsible. The recommendations should be taken into context of good practice of nuclear medicine and do not substitute for national and international legal or regulatory provisions.

## Data Availability

Not applicable.
